# Genetic and Pharmacologic Inhibition of the Neutrophil Elastase Inhibits Experimental Atherosclerosis

**DOI:** 10.1161/JAHA.117.008187

**Published:** 2018-02-08

**Authors:** Guanmei Wen, Weiwei An, Jiangyong Chen, Eithne M. Maguire, Qishan Chen, Feng Yang, Stuart W. A. Pearce, Maria Kyriakides, Li Zhang, Shu Ye, Sussan Nourshargh, Qingzhong Xiao

**Affiliations:** ^1^ Centre for Clinical Pharmacology William Harvey Research Institute Barts and The London School of Medicine and Dentistry Queen Mary University of London London United Kingdom; ^2^ Centre for Microvascular Research William Harvey Research Institute Barts and The London School of Medicine and Dentistry Queen Mary University of London London United Kingdom; ^3^ Key Laboratory of Cardiovascular Diseases The Second Affiliated Hospital School of Basic Medical Sciences Guangzhou Medical University Guangzhou Guangdong China; ^4^ Key Laboratory of Protein Modification and Degradation School of Basic Medical Sciences Guangzhou Medical University Guangzhou Guangdong China; ^5^ Department of Cardiothoracic Surgery Yongchuan Hospital of Chongqing Medical University Chongqing China; ^6^ Department of Cardiology the First Affiliated Hospital School of Medicine Zhejiang University Hangzhou Zhejiang China; ^7^ Department of Cardiovascular Sciences University of Leicester Leicester United Kingdom

**Keywords:** atherosclerosis, ATP‐binding cassette transporter ABCA1, bone marrow transplant, foam cells, GW311616A, inflammation, macrophage, neutrophil elastase, neutrophil elastase inhibitor, protease, Atherosclerosis, Peripheral Vascular Disease, Vascular Disease

## Abstract

**Background:**

To investigate whether neutrophil elastase (NE) plays a causal role in atherosclerosis, and the molecular mechanisms involved.

**Methods and Results:**

NE genetic–deficient mice (Apolipoprotein E^−/−^/NE ^−/−^ mice), bone marrow transplantation, and a specific NE inhibitor (GW311616A) were employed in this study to establish the causal role of NE in atherosclerosis. Aortic expression of NE mRNA and plasma NE activity was significantly increased in high‐fat diet (HFD)–fed wild‐type (WT) (Apolipoprotein E^−/−^) mice but, as expected, not in NE‐deficient mice. Selective NE knockout markedly reduced HFD‐induced atherosclerosis and significantly increased indicators of atherosclerotic plaque stability. While plasma lipid profiles were not affected by NE deficiency, decreased levels of circulating proinflammatory cytokines and inflammatory monocytes (Ly6C^hi^/CD11b^+^) were observed in NE‐deficient mice fed with an HFD for 12 weeks as compared with WT. Bone marrow reconstitution of WT mice with NE ^−/−^ bone marrow cells significantly reduced HFD‐induced atherosclerosis, while bone marrow reconstitution of NE ^−/−^ mice with WT bone marrow cells restored the pathological features of atherosclerotic plaques induced by HFD in NE‐deficient mice. In line with these findings, pharmacological inhibition of NE in WT mice through oral administration of NE inhibitor GW311616A also significantly reduced atherosclerosis. Mechanistically, we demonstrated that NE promotes foam cell formation by increasing ATP‐binding cassette transporter ABCA1 protein degradation and inhibiting macrophage cholesterol efflux.

**Conclusions:**

We outlined a pathogenic role for NE in foam cell formation and atherosclerosis development. Consequently, inhibition of NE may represent a potential therapeutic approach to treating cardiovascular disease.

**Clinical Perspective**

**What Is New?**

- By using 3 different strategies (ie, neutrophil elastase [NE] genetically modified animals, bone marrow transplantation, and an NE selective pharmacologic inhibitor [GW311616A]) in this study, we confirm an important role for NE in atherosclerosis.
- We demonstrate that NE promotes foam cell formation and increases local/systemic inflammation, resulting in atherogenesis.
- We show that NE suppresses cholesterol efflux by enhancing degradation of ATP‐binding cassette transporter ABCA1 protein.

**What Are the Clinical Implications?**

- Our findings on NE in atherogenesis present NE as a novel biomarker for atherosclerosis.
- Oral administration of NE selective pharmacologic inhibitor to correct the dysregulated NE activity and modulate the systemic and local inflammatory environment could serve as a molecular interventional strategy to treat cardiovascular disease.

Neutrophil elastase (NE) is a serine protease, stored in neutrophil azurophilic granules, along with other serine proteases such as cathepsin G (CG) and proteinase 3. NE is stored in azurophilic granules at high concentrations (>5 mmol/L) and is rapidly released into extracellular space in response to various acute and/or chronic inflammatory stimuli. As a major component of neutrophil's armory, NE has been primarily linked to the nonoxidative pathway of intracellular and extracellular pathogen destruction.^1^ Specifically, it has been suggested that NE acts intracellularly within phagolysosomes to digest phagocytosed microorganisms in combination with microbicidal peptides and the membrane‐associated NADPH oxidase system, which produces reactive oxygen metabolites to destroy ingested pathogens.^2^ The antimicrobial role of NE in infection and host defense has been demonstrated in a gene target deletion study where NE‐deficient mice were found to exhibit defective microbicidal activity and were more susceptible than their wild‐type (WT) control littermates to sepsis and death following intraperitoneal challenge with Gram‐negative bacteria.^3^ In addition to this important role in pathogen destruction, NE has potent proteolytic activity on numerous extracellular matrix (ECM) proteins, as well as a variety of nonmatrix proteins such as cytokines/chemokines, cell surface proteins/receptors, and other bioactive soluble proteins.^1^ There is already clear evidence suggesting that NE can cleave and modify certain low‐density lipoprotein (LDL) apolipoproteins, an effect that greatly enhances their uptake by the cellular elements of the arterial wall (particularly macrophages), leading to cellular cholesterol accumulation and foam cell formation.^4^, ^5^ On the other hand, it has been reported that NE also cleaves and modifies antiatherogenic apolipoproteins (high‐density lipoprotein [HDL]), resulting in their rapid degradation by polymorphonuclear cells or macrophages, without triggering cellular cholesterol accumulation.^6^

Apart from neutrophils, freshly isolated monocytes, macrophages, and endothelial cells also express NE,^7^ while its expression in vascular smooth muscle cells (SMCs) remains controversial.^7^, ^8^ As a result of its capacity to efficiently degrade a broad range of ECM proteins and modulate the activity of many inflammatory cytokines and apolipoproteins, NE has been implicated in the pathogenesis of a variety of destructive and inflammatory diseases,^1^ most notably chronic and acute lung diseases such as chronic obstructive pulmonary disease, cystic fibrosis, acute lung injury, and acute respiratory distress syndrome.^2^ However, unlike the intensive investigation into the functional involvements of NE in lung diseases, the significance of NE in cardiovascular diseases has received less attention. Considering the important roles of NE in pathogen destruction and regulation of inflammatory processes, it could be expected that NE plays an important role in atherosclerosis or other inflammation‐driven cardiovascular events. Of note, previous studies have reported abundant amounts of NE within human fibrous and atheromatous plaques, but not in normal arteries,^7^ and a raised plasma NE level has been identified as an independent predictor of cardiovascular events in patients with angina pectoris.^9^ Furthermore, serum elastase activity is associated with systolic hypertension and arterial stiffness.^10^ Despite these studies, it is unknown whether NE plays a causal role in atherogenesis or is simply a bystanding marker in the abovementioned disease settings. In the current study, by generating NE and Apolipoprotein E (ApoE) double knockout mice, and utilizing a highly potent and selective NE inhibitor, we provide direct evidence to support a causal role for NE in atherosclerosis.

## Materials and Methods

The data that support the findings of this study are available from the corresponding author upon reasonable request.

### Materials

Antibodies against NE (ab68672), GAPDH (ab8245), ABCA1 (ab18180), ABCG1 (ab52617), SR‐B1 (ab217318), CD68 (ab201845, ab125212 or ab955), Ly6 g (Gr1) [RB6‐8C5] (ab25024, FITC‐conjugated), Ly6 g [1A8] (ab210204), Ly6c [ER‐MP 20] (ab15686, FITC‐conjugated), CD11b [M1/70] (ab25533, PE/Cy5‐conjugated), and CD31 (ab25644, PE‐conjugated) were purchased from Abcam. The antibody against CD45 (103105, PE‐conjugated) was from Biolegend UK. Antibodies against α‐tubulin (mouse) and smooth muscle α‐actin (Clone 1A4, A5228) were from Sigma. All secondary antibodies were from Sigma. All other materials used in this study were purchased from Sigma except those otherwise specified.

### Animals and Atherosclerosis

All animal experiments were performed according to the Animals (Scientific Procedures) Act 1986 (United Kingdom), and all protocols were approved by Queen Mary, University of London, ethics review board (project number: 70/7216). In addition, the principles governing the care and treatment of animals, as stated in the *Guide for the Care and Use of Laboratory Animals* published by the National Academy of Sciences (8th ed, 2011), were followed at all times during this study. All mice were euthanized by placing them under deep anesthesia with 100% O2/5% isoflurane, followed by decapitation.

NE^−/−^/ApoE^+/+^ mice (Elane^−/−^ mice from The Jackson Laboratory, 006112)^11^, ^12^ (C57BL/6 background) that were backcrossed with C57BL/6 mice for at least 10 generations were crossbred with NE^+/+^/ApoE^−/−^ mice (C57BL/6 background)^13^, ^14^ to generate NE^+/−^/ApoE^+/−^ double heterozygous mice. NE^+/−^/ApoE^+/−^ double heterozygous mice were bred to produce NE^−/−^/ApoE^−/−^ double knockout (NE_KO) mice and NE^+/+^/ApoE^−/−^ WT controls (WT mice: littermates of NE_KO). Genotyping was performed using the protocol provided by The Jackson Laboratory (Bar Harbor). Eight‐week‐old male mice were fed a high‐fat diet (HFD) containing 21% fat, 1.25% cholesterol, and 0% cholate (AIN‐76A/Clinton‐Cybulsky Cholesterol Series #3‐108, T‐58R6‐1810021, Test Diet Limited) for the indicated durations to induce atherosclerosis.

### Bone Marrow Transplantation

To induce bone marrow aplasia, 8‐week‐old male recipient mice (WT or NE_KO) were exposed to a single dose of 10 Gy x‐ray total body irradiation 24 hours before the bone marrow transplant (BMT). Bone marrow cell suspensions were isolated by flushing the femurs and tibias from WT or NE_KO mice with phosphate‐buffered saline. Single‐cell suspensions were prepared by passing the cells through a 30‐μm nylon gauze. Irradiated recipients received 1.5×10^7^ bone marrow cells by intravenous injection into the tail vein. Polymerase chain reaction detection of NE genotype was performed on DNA extracted from blood of recipient mice 4 to 5 weeks after BMT to confirm the efficiency of this BMT technique. Recipient mice with correct bone marrow cell reconstitution were fed an HFD for 12 weeks to induce atherosclerosis. Bone marrow reconstitution of recipient mice was further confirmed by polymerase chain reaction analyses of NE mRNA expression levels in peripheral blood monocytes at the end of procedure.

### Oral Administration of GW311616A

GW311616A^15^ has been developed as a highly potent, selective, intracellular, orally bioavailable, and long duration human NE (HNE) inhibitor. In the previous study,^15^ it was reported that a single oral dose of 2 mg/kg GW311616A can rapidly abolish circulating NE activity, with >90% NE activity inhibition being maintained for 4 days. Accordingly, a dose of 2 mg/kg GW311616A was used in this study to pharmacologically inhibit the NE activity in mice fed an HFD. Specifically, 8‐week‐old male WT mice were fed an HFD for 6 weeks, and GW311616A (Sigma‐Aldrich, G8419) or vehicle was randomly administered (2 mg/kg by gavage; twice a week) into WT (NE^+/+^/ApoE^−/−^) mice from week 7 to week 12 of HFD.

### Characterization of Atherosclerotic Lesions

The vascular tree and heart were carefully isolated and the atherosclerotic lesions were characterized as described in previous studies.^13^, ^14^, ^16^ Briefly, after euthanization, the aorta, from heart to the level below the iliac bifurcation, was extensively exposed. Perivascular connective tissue and adipose tissue around the aorta and the major artery branches were carefully removed using fine iris scissors and delicate forceps. The whole vascular tree along with heart was harvested. The heart harboring the aortic roots was carefully cut from the level above the coronary artery at the base of the heart and snap‐frozen in liquid nitrogen immediately for later use. The rest of the aortas were cut longitudinally, fixed in 4% paraformaldehyde, and stained with Oil Red “O” (Sigma‐Aldrich) to visualize the atherosclerotic plaque area (fatty streaks). The lesion area was calculated using Image‐Pro Plus 6.0 software (Media Cybernetics).

The extent of atherosclerotic lesions of aortic roots, lipid accumulation, and collagen content within atherosclerotic lesions in WT or NE_KO mice were analyzed by hematoxylin/eosin (H&E), Oil Red “O”, and Sirius Red staining, respectively. The stained sections or aortas were photographed using a digital camera. The area of intima and media–, Oil Red “O” (refer to lipid accumulation)–, or Sirius Red (refer to collagen content)–stained area in a given image was highlighted and quantified (μm^2^ for media and lesion size; percentage over the atherosclerotic lesion area for lipid accumulation and collagen content) by computer‐assisted quantification using Axiovision software. Two experienced investigators blinded to the treatments each tissue had received performed this analysis. Three (6 to 8 sections for H&E staining) sections were analyzed per aortic roots (or per mouse) and averaged. In some sections with H&E staining, the acellular area within atherosclerotic plaques were highlighted and quantified as necrotic cores (percentage over the atherosclerotic lesion area).

### Immunofluorescent Staining of the Frozen Tissue Sections

Immunofluorescent assay was performed as previously described.^13^, ^14^, ^17^, ^18^, ^19^ Briefly, frozen tissue sections were labeled with appropriate isotype IgG control or antibodies as indicated in the figures and visualized with appropriate secondary antibodies conjugated with CF 488A or CF 568 (Sigma) for the unconjugated primary antibodies. Sections were counterstained with 4′,6‐diamidino‐2‐phenylindole (DAPI; Sigma). Images were examined and acquired with SP5 confocal microscopy with Leica TCS Sp5 software (Leica) at room temperature. Positively stained areas for the respective antibody in a given image were highlighted and quantified (percentage over the atherosclerotic lesion area) by computer‐assisted quantification using the Axiovision software. Two experienced investigators blinded to the treatments each tissue had received performed this analysis. Three sections were analyzed per aortic roots (or per mouse) and averaged.

### Semiquantitative Analysis of Lipid‐Loading and ABCA1 Protein Expression of Lesion Macrophages

To analyze the lipid loading on lesion macrophages, frozen sections were fixed with 4% paraformaldehyde for 5 minutes and rinsed in PBS 3 times (3×10 minutes). Subsequently, sections were incubated with BODIPY 493/503 (D3922, ThermoFisher, 5 μmol/L in DMSO) at room temperature for 30 minutes. After being rinsed in PBS 3 times, sections were subjected to a regular immunofluorescent staining with CD68 antibody. Standard double immunofluorescent staining with antibodies against CD68 and ABCA1 was conducted with frozen aortic roots sections as previously described to detect ABCA1 protein expression levels on macrophages within atherosclerotic plaques. Images were examined and acquired with SP5 confocal microscopy with Leica TCS Sp5 software at room temperature. Ten atherosclerotic lesion macrophages (CD68^+^ cells) in a given image were randomly highlighted, and mean fluorescence intensity of ABCA1 or Bodipy staining as well as DAPI for each randomly selected macrophage were measured using Image‐Pro Plus 6.0 software (Media Cybernetics). Relative mean fluorescence intensity of ABCA1 or Bodipy staining against DAPI staining were calculated and presented. Two experienced investigators blinded to the treatments conducted this analysis. Three sections were analyzed per aortic roots (or per mouse) and averaged.

### Arterial Macrophage Isolation and Characterization

Arterial tree including ascending aorta, aorta arch, and descending aorta were harvested from mice fed an HFD for 12 weeks. Perivascular connective tissue and adipose tissue around the aorta were carefully removed using fine iris scissors and delicate forceps. The aortas were digested with 0.25 mg/mL Liberase (Liberase TM Research Grade with medium Thermolysin concentration, Sigma) in RPMI1640 medium supplemented with 10% FCS at 37°C for 1 hour. The obtained cell suspensions were filtered through a 40‐μm strainer and subsequently incubated with rat IgG on ice for 30 minutes. Subsequently, cells were washed and incubated with anti‐CD68 antibody [FA‐11] (Alexa Fluor 647) (ab201845, Abcam) for 30 minutes at room temperature and then washed twice. CD68^+^ cells were sorted using BD FACSAria II and prepared for cell smears. The purity of the sorted macrophages was higher than 90% and the estimated yield of macrophages per aorta (mouse) were (1.5±0.36)×10^5^ for WT mice and (0.78±0.37)×10^5^ for NE_KO mice, respectively. The sorted macrophages were subjected to a standard immunofluorescent staining with antibody against ABCA1 and Oil Red “O” staining, followed by semiquantitative analysis using Image‐Pro Plus 6.0 software to examine ABCA1 protein expression levels and the lipid accumulation in arterial lesion macrophages, as described in other sections.

### Plasma Cholesterol and Triglyceride Analysis

After an overnight fasting period, ≈500 to 800 μL of peripheral blood was drawn from each individual mouse through the right atrium of the heart before perfusion. Plasma total cholesterol (ab65359, Abcam), triglycerides (ab65336, Abcam), HDL cholesterol, and LDL cholesterol/very LDL fraction (HDL and LDL/very LDL Fraction Assay Kit, ab65390, Abcam) were measured by colorimetric assay according to the manufacturer's instructions. LDL‐Cholesterol Assay Kit (#80069) from Crystal Chem was used for measuring plasma LDL cholesterol according to the protocol provided in the kit.

### Plasma Cytokines

Plasma interleukin (IL)‐8 was assessed using IL8 ELISA Kit (ABIN2535647, detection limit: 2 pg/mL) from Antibody online, while the levels of other cytokines in the plasma were measured using their respective ELISA kit (88‐7013‐22 for IL‐1β [8 pg/mL]; EM2IL6 for IL‐6 [7 pg/mL]; EMIL12B for IL‐12 [5 pg/mL]; EMMCP1 for monocyte chemoattractant protein‐1 [4 pg/mL]; BMS607‐3 for tumor necrosis factor α (TNFα; 3.7 pg/mL]; and KMC4021 for interferon γ (IFN‐γ) [<2 pg/mL]) purchased from ThermoFisher, according to the manufacturer's instructions.

### Plasma NE Activity and NE Protein Level in the BMM–Derived Conditioned Culture Medium

Plasma NE activity was determined using N‐methoxysuccinyl‐Ala‐Ala‐Pro‐Val‐p‐nitroanilide (M4765, Sigma), a highly specific synthetic substrate for NE according to the method described in previous studies.^20^, ^21^, ^22^ Briefly, a 100‐μL plasma sample was incubated with 0.1 M Tris–HCl buffer (pH 8.0) containing 0.5 M NaCl and 1 mmol/L substrate in a final volume of 1.0 ml at 37°C for 24 hours. The amount of p‐nitroanilide liberated from the substrate by NE was measured spectrophotometrically at 405 nm and calculated from a standard curve of p‐nitroanilide (185310, Sigma). One unit of NE activity was defined as the quantity of enzyme that liberated 10 μmol/L of p‐nitroanilide in 24 hours. NE protein level in the bone marrow macrophage (BMM)–derived conditioned culture medium was measured using an ELISA kit purchased from R&D System (DY4517‐05, detection limit: 12.50 pg/mL), according to the manufacturer's instructions.

### Plasma CG, Trypsin, and Plasmin Activity

Plasma CG, trypsin, and plasmin activity were determined using their respective commercially available kits by following the instructions provided with the kits: Cathepsin G Activity Assay Kit (Colorimetric) (ab126780; Abcam, UK), Trypsin Activity Colorimetric Assay Kit (MAK290‐1KT; Sigma, UK), or Plasmin Activity Assay Kit (Fluorometric) (MAK244‐1KT; Sigma, UK).

### Foam Cell Formation Assay

For in vitro foam cell formation analyses, bone marrow mononuclear cells isolated from WT or NE_KO mice were differentiated into BMMs as described in our previous study,^23^ followed by an incubation with 5 μg/mL of DiI‐Ac‐LDL (10574053, Fisher Scientific) for 5 hours. The cells were fixed with paraformaldehyde and counterstained with DAPI. Images were randomly taken, and the ratio of mean fluorescence intensity of DiI‐Ac‐LDL (red) to that of DAPI (blue) were analyzed.

For in vivo foam cell formation assays, a method described in the previous studies^24^, ^25^ was used. Briefly, 8‐week‐old WT and NE_KO male mice (n=5 per group) were placed on an HFD for 12 weeks. Thioglycollate (3%) was injected via IP method, and 3 days later peritoneal macrophages were isolated, plated, and stained with Oil Red “O.” Three images were randomly taken from each mouse, and the integrated optical density per cell for Oil Red “O” staining were analyzed.

### DiI‐Ac‐LDL Binding Assay

DiI‐Ac‐LDL binding assays were conducted as described in a previous study.^26^ Briefly, BMMs or peritoneal‐naive macrophages were isolated from WT and NE_KO mice and incubated with 10 μg/mL DiI‐Ac‐LDL in the absence or presence of 1 μg/mL of active HNE (Abcam, ab91099) for 3 to 4 hours at 4°C. Cells were washed and lysates were analyzed by fluorometry with a 540‐nm excitation laser line and 590‐nm emission filters.

### Macrophage Cholesterol Efflux Assay

A Cholesterol Efflux Assay Kit (MAK192‐1KT) from Sigma was used to detect the macrophage cholesterol efflux capacity according to the procedure provided in the kit. Briefly, the same number of BMMs or peritoneal‐naive macrophages isolated from WT and NE_KO mice were plated into a 96‐well plate. Cells were incubated at 37°C with 5% CO2 for 2 hours, followed by a wash with RPMI‐1640 medium without serum. After equilibration, cells were incubated with 10 μg/mL of Apolipoprotein A‐I (ApoA1, 73366, Sigma) or 50 μg/mL of HDL (L1567, Sigma) for 4 to 6 hours. Cell supernatants were harvested and adherent cells were solubilized using cell lysis buffer. The fluorescent intensity of the medium (Fm) and cell lysate (Fc) was measured by fluorometry (Molecular Devices) with a 482‐nm excitation laser line and 515‐nm emission filters. The percent cholesterol efflux presented in the samples (C) was determined with the equation [C=100%×Fm/(Fm+Fc)].

### Real‐Time Quantitative Polymerase Chain Reaction

Real‐time quantitative polymerase chain reaction was performed as previously described.^17^, ^18^, ^19^, ^27^, ^28^, ^29^, ^30^, ^31^ Briefly, total RNA was extracted from murine aortas or cells using Trizol reagent (Sigma) according to the manufacturer's instructions and subjected to DNase I (Sigma) digestion to remove potential DNA contamination. Reverse transcription for long RNAs was performed using an Improm‐II Reverse Transciption Kit (Promega) with RNase inhibitor (Promega) and Random primers (Promega). The resultant cDNA was diluted to a working concentration of 5 ng/μL and stored at −20°C. NCode KAPA SYBR FAST ABI Prism qPCR Syber green SuperMix (Sigma) was used to quantify cDNA levels. Relative mRNA expression level was defined as the ratio of target gene expression level to 18S expression level, with that of the control sample set as 1.0. Primers were designed using Primer Express software (Applied Biosystems) and the sequence for each primer is listed in the Table.

**Table 1:** Primer Sets Used in the Present Study

Gene Names	Forward (5′–3′)	Reverse (5′–3′)	Application
18s	AAACGGCTACCACATCCAAG	CCTCCAATGGATCCTCGTTA	RT‐qPCR
NE	CAGAGGCGTGGAGGTCATTT	CTACCTGCACTGACCGGAAA	RT‐qPCR
PR3	TCCTATGCCGGGAACACAAC	CGGATCACGAAGGAGTCCAC	RT‐qPCR
CG	GCCATCCGCCATCCTGATTA	ATCCCCTGGCTGCAGTTTTT	RT‐qPCR
TNFα	AGGCACTCCCCCAAAAGATG	TGAGGGTCTGGGCCATAGAA	RT‐qPCR
IFN‐γ	AGCAAGGCGAAAAAGGATGC	TCATTGAATGCTTGGCGCTG	RT‐qPCR
MCP‐1	CCCCAAGAAGGAATGGGTCC	TGCTTGAGGTGGTTGTGGAA	RT‐qPCR
IL‐6	GTGGCTAAGGACCAAGACCA	TAACGCACTAGGTTTGCCGA	RT‐qPCR
IL‐12	AGTGACATGTGGAATGGCGT	CAGTTCAATGGGCAGGGTCT	RT‐qPCR
IL‐1β	TGCCACCTTTTGACAGTGATG	TGATGTGCTGCTGCGAGATT	RT‐qPCR
IL‐8	CCTGATGCTCCATGGGTGAA	ACAGAAGCTTCATTGCCGGT	RT‐qPCR
ABCA1	CGACCATGAAAGTGACACGC	AGCACATAGGTCAGCTCGTG	RT‐qPCR

CG indicates cathepsin G; IFN‐γ, interferon γ; IL, interleukin; MCP‐1, monocyte chemoattractant protein‐1; NE, neutrophil elastase; PR3, proteinase 3; RT‐qPCR, real‐time quantitative polymerase chain reaction; TNFα, tumor necrosis factor α.

### Immunoblotting

Cells were harvested and lysed in a lysis buffer (50 mmol/L Tris‐Cl pH 7.5, 150 mmol/L NaCl, 1 mmol/L EDTA pH 8.0) supplemented with protease inhibitors and 0.5% Triton by sonication. Forty micrograms of protein were separated by SDS‐PAGE with 4% to 20% Tris‐Glycine gel (Invitrogen) and subjected to standard Western blot analysis. In some experiments, the blots were subjected to densitometric analysis with ImageJ software. ABCA1 relative protein expression level was defined as the ratio of ABCA1 protein expression level to GAPDH or α‐tubulin expression level with that of the control sample (WT, 0 hour, or vehicle control for inhibitors, respectively) set as 1.0.

### Flow Cytometry Assay

Bone marrow and peripheral blood nucleated cells (0.5–1.0×10^6^) isolated from WT or NE_KO mice were incubated for 60 minutes on ice with various antibodies (1 μg/mL), as indicated in the figures, or an isotype‐matched control antibody (1 μg/mL). Subsequently, the cells were fixed in 1% paraformaldehyde and then analyzed by flow cytometry.

### Statistical Analysis

Data were expressed as mean±SEM except those otherwise specified in the figures. Statistical analysis was performed using GraphPad Prism 5. Shapiro‐Wilk normality test was used for checking the normality of the data. Data with a Shapiro‐Wilk test *P*>0.05 were considered to fit a normal distribution. Two‐tailed unpaired Student *t* test was used for comparisons between 2 groups, or 1 (2)‐way ANOVA test with Bonferroni post hoc test was applied when more than 2 groups were compared if the data displayed a normal distribution. Conversely, nonparametric Mann–Whitney *U* test or Kruskal–Wallis 1‐way ANOVA with a post hoc test of Dunn test was applied for comparing 2 groups and ≥3 groups, respectively, if the data did not display normal distribution. α=0.05 was chosen as the significance level, and a value of *P*<0.05 was considered statistically significant.

## Results

### Development of Atherosclerotic Lesions Is Associated With Increased NE Expression at mRNA and Protein Level and Plasma NE Activity

**Figure 1:**
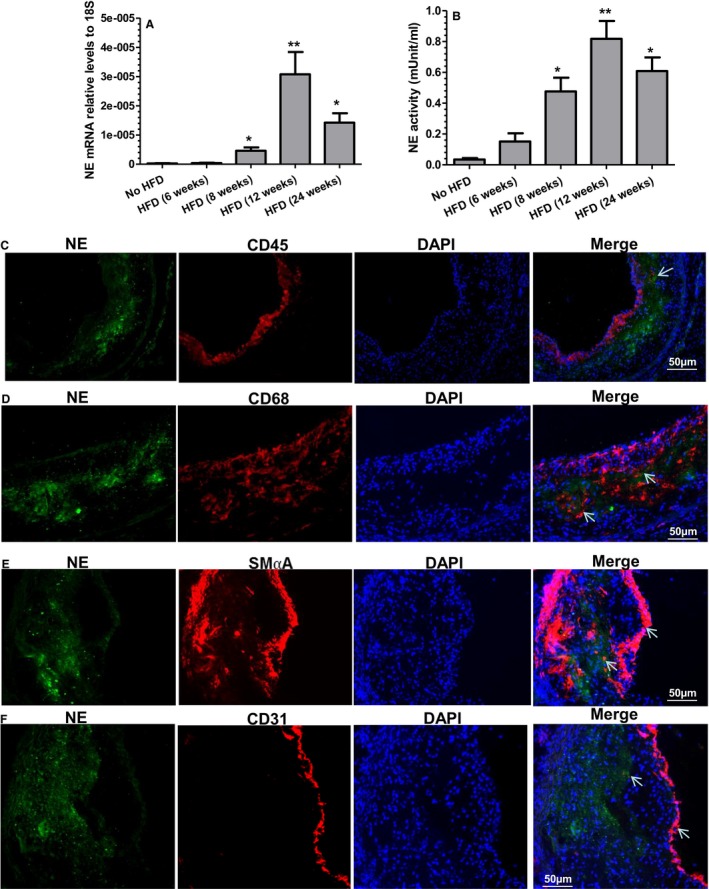
Aortic expression of neutrophil elastase (NE) and circulating NE activity during atherosclerosis, as well as NE expression within atherosclerotic plaques. A and B, Increased aortic NE gene expression and circulating NE activity during atherosclerosis development. Eight‐week‐old male Apolipoprotein E (ApoE) knockout (ApoE^−/−^) mice were fed a high‐fat diet (HFD) for 6, 8, 12, or 24 weeks, respectively. Aortas and plasma were harvested. Total RNAs were exacted from aortas and subjected to real‐time quantitative polymerase chain reaction analyses to examine NE mRNA expression levels in aortas (A). Plasma NE activity was determined using a synthetic substrate, N‐methoxysuccinyl‐Ala‐Ala‐Pro‐Val p‐nitroanilide (B). Twenty‐week‐old male ApoE^−/−^ mice placed on normal chow diet were used as a control (no HFD). Data presented are the average of 5 mice for each group (n=5 mice). *P<0.05, **P<0.01 (vs no HFD). C through F, NE protein detection in atherosclerotic lesions. Eight‐week‐old ApoE^−/−^ mice were fed an HFD for 12 weeks. Aortic roots were harvested, sectioned, and subjected to double immunofluorescent staining analyses using antibodies against NE and CD45 (C), CD68 (D), smooth muscle α‐actin (E), or CD31 (F), respectively. Respective representative images from 5 mice (n=5 mice) are presented. White arrows indicate the weak colocalization of NE with different cells within plaques. DAPI indicates 4′,6‐diamidino‐2‐phenylindole.

**Figure 2:**
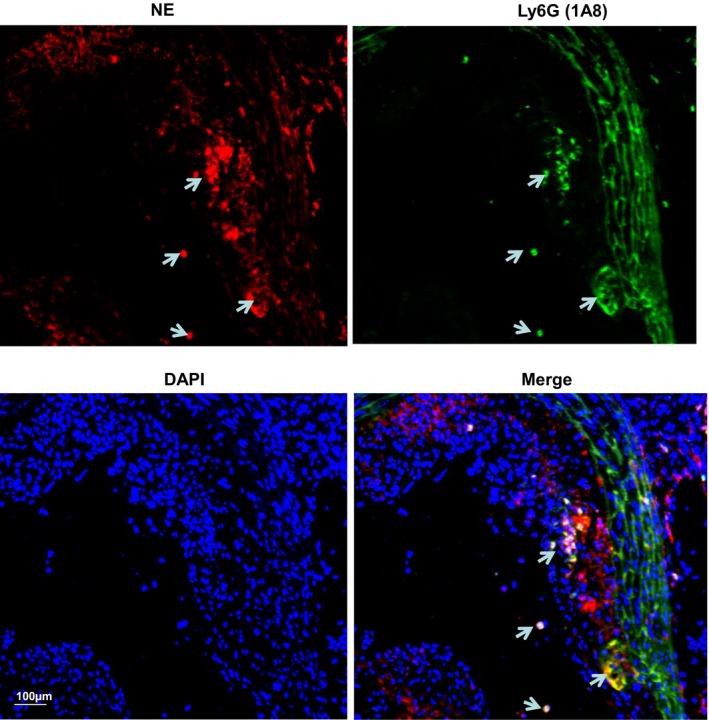
Atherosclerotic lesion neutrophils express neutrophil elastase (NE). Eight‐week‐old Apolipoprotein E^−/−^ mice were fed a high‐fat diet for 12 weeks. Aortic roots were harvested, sectioned, and subjected to double immunofluorescent staining analyses using antibodies against NE and Ly6G (1A8). Respective representative images from 5 mice (n=5 mice) are presented. White arrows indicate the Ly6G^+^ neutrophils within plaques express NE. DAPI indicates 4′,6‐diamidino‐2‐phenylindole.

To explore the potential function of NE in atherosclerosis, 8‐week‐old ApoE single knockout male mice were fed a Western diet (HFD) for 6, 8, 12, and 24 weeks, respectively. Compared with mice placed on normal chow diet, a trend of increased NE mRNA expression was seen at 6 weeks, and a significant elevation in aortic NE mRNA expression level was found in all other groups (8, 12, and 24 weeks) (Figure 1A). Circulating NE activity (Figure 1B) was observed to be elevated in mice fed an HFD from 6 weeks and significantly increased in all other groups tested (8, 12, and 24 weeks). Following HFD, both plasma NE activity and aortic NE expression levels were further increased and peaked in mice fed an HFD for 12 weeks (Figure 1A and 1B). Double immunofluorescence staining of atherosclerotic lesions with antibodies against NE and other cell markers showed weak colocalization of NE with CD45 (leukocytes)‐, CD‐68 (macrophages)–, smooth muscle α‐actin (SMCs)–, and CD31 (endothelial cells)‐expressing cells, but the majority of NE was localized within the ECM in plaques (Figure 1C through 1F). Unsurprisingly, although the neutrophils were difficult to detect within atherosclerotic plaques, all the detected lesion Ly6G^+^ neutrophils showed strong positive expression for NE staining (Figure 2).

### NE Deficiency Results in Reduced Atherosclerosis

**Figure 3:**
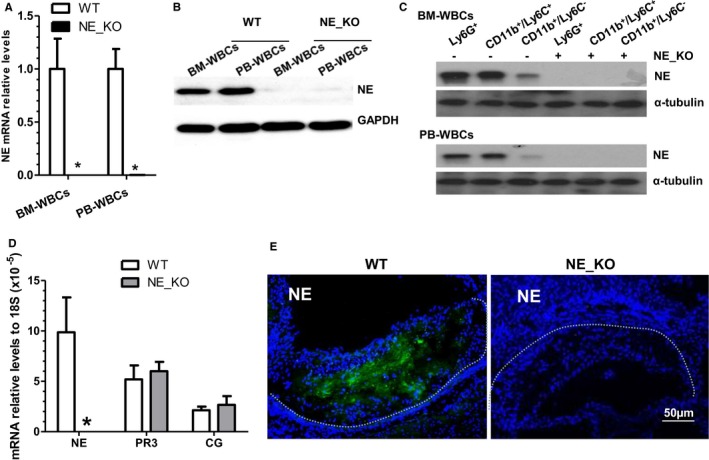
Validation of neutrophil elastase (NE) gene inactivation and detection of other neutrophil serine proteases in NE^−/−^/Apolipoprotein E (ApoE)^−/−^ double knockout (NE_KO) mice. A through B, Neither NE mRNA nor NE protein was detected in NE‐deficient bone marrow white blood cells (BM‐WBCs) and peripheral blood white blood cells (PB‐WBCs) (leukocytes). Total RNAs and proteins were harvested from BM‐WBCs or PB‐WBCs of wild‐type (WT) (ApoE^−/−^/NE ^+/+^) and NE_KO (ApoE^−/−^/NE ^−/−^) mice and subjected to real‐time quantitative polymerase chain reaction (RT‐qPCR) and Western blot analyses, respectively. Data presented are averages or representative images of 5 mice within each group (n=5 mice). *P<0.05 (vs WT mice). C, Both neutrophils and monocytes express NE. Neutrophils (Ly6G^+^ cells) were sorted from BM‐WBCs and PB‐WBCs isolated from WT and NE_KO mice using an anti‐mouse Ly‐6G MicroBead Kit (130‐092‐332, Miltenyi Biotec). CD11b^+^/Ly6C^+^ and CD11b^+^/Ly6C^−^ cells were further sorted from the remaining Ly6G^−^ cells by staining them with antibodies against CD11b and Ly6C. Protein was extracted from the sorted cells and subjected to standard Western blotting analysis using NE antibody. Data presented are representative images of 5 mice within each group (n=5 mice). D, Aortic mRNA expression of NE, proteinase 3, and cathepsin G. Eight‐week‐old male WT and NE_KO mice were fed a high‐fat diet (HFD) for 12 weeks. Total RNAs were extracted from aortas of the WT and NE_KO mice and subjected to RT‐qPCR analyses. E, NE is undetectable within the atherosclerotic plaques of NE_KO mice. Aortic roots harvested from both male WT and NE_KO mice fed an HFD for 12 weeks were sectioned and subjected to immunofluorescence staining with an NE antibody. No clear fluorescence signal was observed within the atherosclerotic plaque of NE‐KO mice. Data presented in (D and E) are averages or representative images of 5 mice for each group (n=5 mice). *P<0.05 (vs WT mice). Dot lines indicate the boundary between media layer and atherosclerotic lesion.

**Figure 4:**
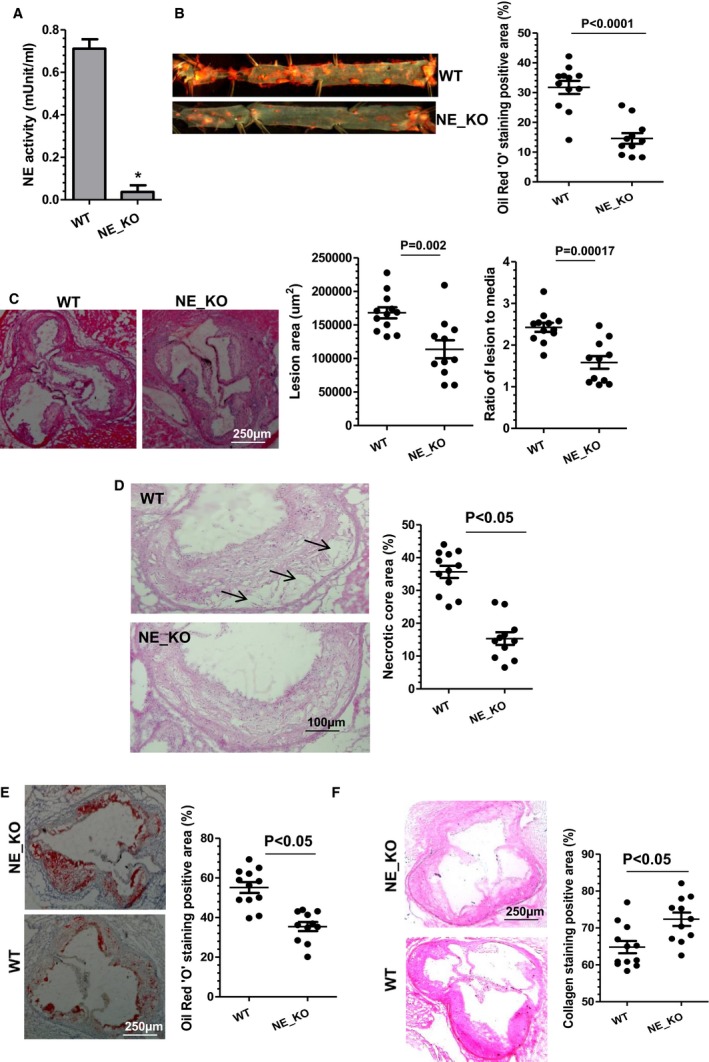
Reduced atherosclerosis in neutrophil elastase (NE)^−/−^/Apolipoprotein E (ApoE)^−/−^ mice. Both wild‐type (WT mice: NE ^+/+^/ApoE^−/−^) and NE gene knockout (NE_KO mice: NE ^−/−^/ApoE^−/−^) male mice were fed a high‐fat diet for 12 weeks. Plasma (A), aortas (B), and aortic roots (C through F) from WT and NE_KO mice were harvested and subjected to plasma NE activity assay (A), Oil Red “O” enface staining (B), hematoxylin‐eosin staining (C and D), Oil Red “O” staining (E), and collagen staining with Sirius Red (F), respectively. Respective representative image and quantitative data from 12 WT and 11 NE_KO mice, respectively, are presented. *P<0.05 (vs WT mice).

To investigate whether NE plays a causal role in atherosclerosis, NE_KO mice were generated and, together with their control littermates (WT mice, ApoE single knockout mice), were fed an HFD for 12 weeks to induce atherosclerosis. No NE mRNA (Figure 3A) or protein (Figure 3B) expression was detected in bone marrow or circulating leukocytes isolated from NE‐deficient mice, validating this genetically modified model. As expected, both neutrophils (Ly6G^+^ cells) and inflammatory monocytes (Ly6G^−^/CD11b^+^/Ly6C^+^ cells) in bone marrow or peripheral blood expressed a comparably high level of NE protein, while “alternative” or “patrolling” monocytes (Ly6G^−^/CD11b^+^/Ly6C^−^ cells) expressed no or a very limited amount of NE protein (Figure 3C). Conversely, no NE protein was detected in the respective cell populations isolated from NE_KO mice, further validating the NE genetically modified model. Moreover, NE gene expression could not be detected in aortas isolated from NE‐deficient mice fed an HFD for 12 weeks (Figure 3D), no NE protein was detected within the atherosclerotic lesions of NE gene knockout mice (Figure 3E), and an almost undetectably low level of plasma NE activity was observed in these mice (Figure 4A). By contrast, no significant difference was observed in gene expression levels of 2 other mouse neutrophil serine proteases, CG, and proteinase 3 in the aorta of WT and NE_KO mice fed an HFD for 12 weeks (Figure 3D).

**Figure 5:**
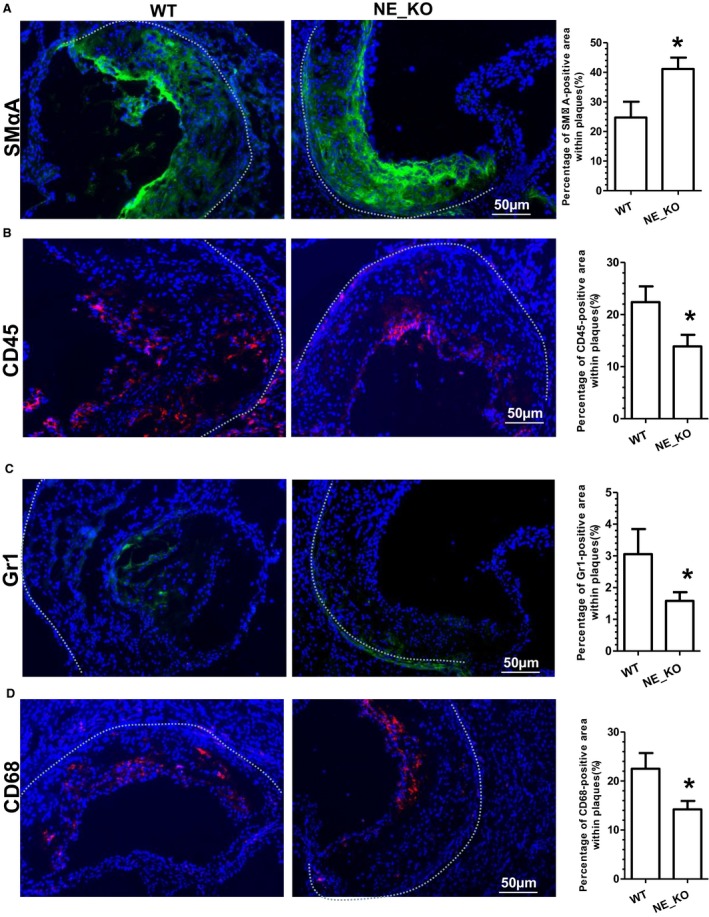
Neutrophil elastase (NE) gene inactivation results in a stable atherosclerotic phenotype. Both wild‐type (WT) and NE^−/−^/Apoliporotein E^−/−^ double knockout (NE_KO) male mice were fed a high‐fat diet for 12 weeks. Aortic roots from WT and NE_KO mice were harvested and subjected to immunofluorescent staining analyses using antibodies against smooth muscle α‐actin (SMαA) (A), CD45 (B), Gr1 (C), or CD68 (D), respectively. Respective representative images and quantitative data from 12 WT and 11 NE_KO mice, respectively, are presented. Dot lines indicate the boundary between media layer and atherosclerotic lesion. *P<0.05 (vs WT mice).

Results from aortic tree enface Oil Red “O” staining showed that extensive aortic atherosclerotic lesions were observed in control WT mice fed an HFD (Figure 4B). In contrast, NE_KO mice had substantially less aortic atherosclerosis (>50% reduction compared with controls, *P*<0.0001) (Figure 4B). Such a reduction was also observed in aortic roots of NE_KO mice (Figure 4C). Compared with atherosclerotic plaques in WT mice, those in NE_KO mice had significantly smaller necrotic cores (Figure 4D) and less lipid accumulation (Figure 4E) but exhibited a much higher collagen content (Figure 4F). Immunofluorescence staining showed that atherosclerotic plaques of NE_KO mice had a much higher level of SMC content (Figure 5A) while containing fewer leukocytes (Figure 5B), most notably neutrophils (Figure 5C) and macrophages (Figure 5D).

### Systemic and Local Aortic Inflammation Is Diminished in NE_KO Mice Fed an HFD

**Figure 6:**
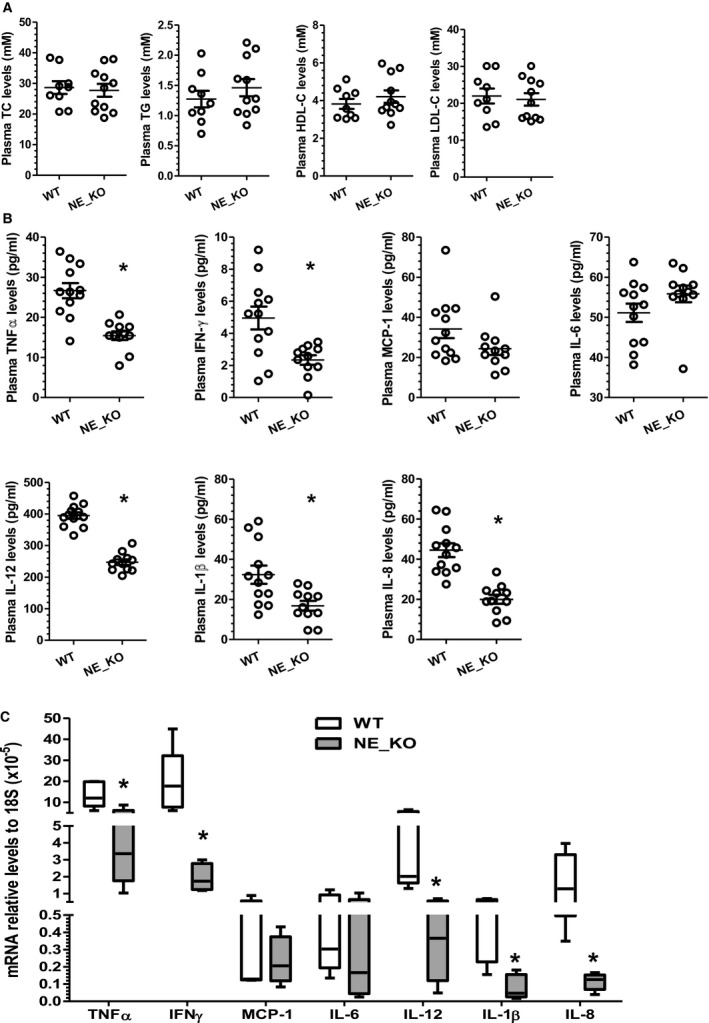
Systemic and local inflammation is diminished in neutrophil elastase^−/−^/Apolipoprotein E^−/−^ double knockout (NE_KO) mice fed a high‐fat diet (HFD) for 12 weeks. Both wild‐type (WT) and NE_KO male mice were fed an HFD for 12 weeks. Plasma (A and B) and aortas (C) were harvested and subjected to plasma lipid (A) and inflammatory cytokine (B) profile assay, as well as real‐time quantitative polymerase chain reaction analyses to detect aortic (local) expression of inflammatory genes as indicated (C), respectively. Quantitative data (mean±SEM) for both plasma lipid and inflammatory cytokine levels from 12 WT and 11 NE_KO mice are presented. *P<0.05 (vs WT mice, unpaired *t* test). Data in C were aortic mRNA expression levels from 5 mice (n=5 mice per group) presented as median with interquartile range. *P<0.05 (vs WT mice, Mann–Whitney *U* Test). HDL‐C indicates high‐density lipoprotein cholesterol; IFN‐γ, interferon γ; IL, interleukin; LDL‐C, low‐density lipoprotein cholesterol; MCP‐1, monocyte chemoattractant protein‐1; TC, total cholesterol; TG, triglyceride; TNFα, tumor necrosis factor α.

**Figure 7:**
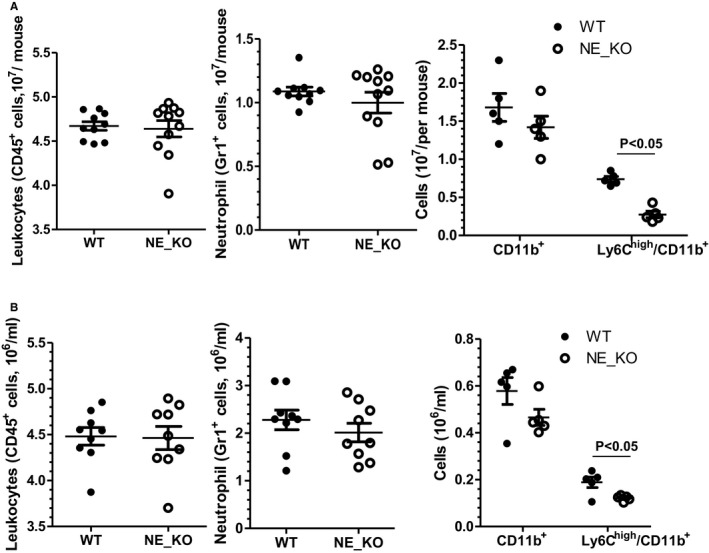
Neutrophil elastase (NE) deficiency results in a decreased level of circulating inflammatory monocytes. Nucleated cells were isolated from bone marrow (A) or peripheral blood (B) of WT and NE^−/−^/Apolipoprotein E (ApoE)^−/−^ double knockout (NE_KO) male mice fed a high‐fat diet for 12 weeks and subjected to flow cytometry analyses with antibodies against neutrophil (CD45 and Gr‐1) and monocyte (CD11b and Ly6C) markers, respectively. Black lines represent mean±SEM of each cell population from WT and NE_KO mice, respectively. Each circle indicates the individual measurement for each mouse.

There was no significant difference in plasma total cholesterol, triglycerides, HDL cholesterol, and LDL cholesterol between WT and NE_KO mice (Figure 6A). However, significantly lower levels of plasma proinflammatory cytokines (such as TNFα, IFN‐γ, IL‐1β, IL‐8, and IL‐12) were observed in NE_KO mice (Figure 6B). Moreover, NE gene inactivation resulted in a trend towards decreased and increased plasma monocyte chemoattractant protein‐1 and IL‐6, respectively (Figure 6B). Furthermore, compared with that of controls, a much lower aortic mRNA expression level of TNFα, IFN‐γ, IL‐1β, IL‐8, and IL‐12 were detected in NE_KO mice fed an HFD for 12 weeks (Figure 6C). In assessing the contribution of leukocytes to the above findings, flow cytometry analyses showed no significant difference in the number of bone marrow (Figure 7A) or circulating (Figure 7B) leukocytes (CD45‐positive cells) and neutrophils (Gr1‐positive cells) between WT and NE_KO mice. Of interest, however, a significant decrease in bone marrow and circulating inflammatory monocyte level (Ly6C high and CD11b‐positive cells), but not total monocyte (CD11b‐positive cells) level, was observed in NE_KO mice (Figure 7).

### Bone Marrow Cell–Derived NE Plays a Major Role in Atherosclerotic Lesion Formation

**Figure 8:**
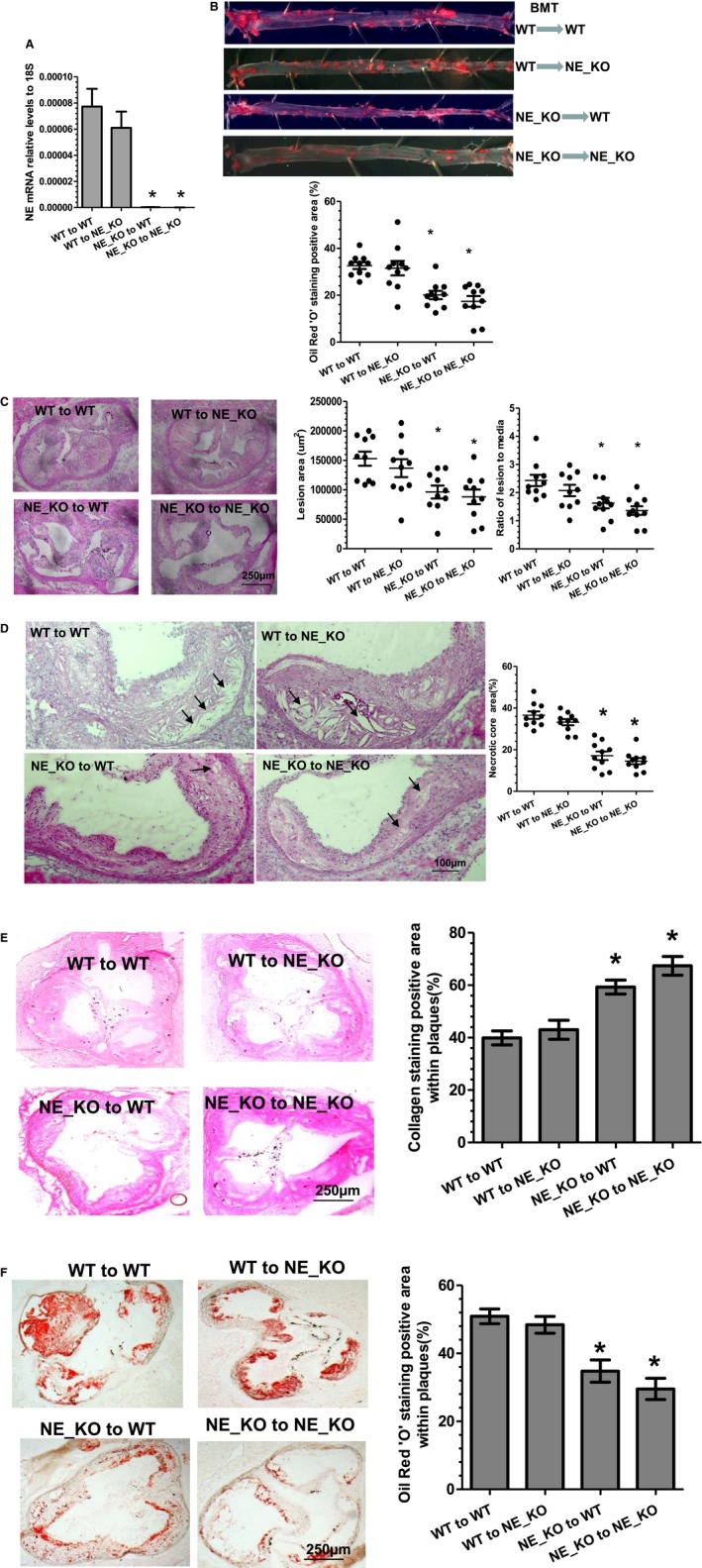
Bone marrow cell–derived neutrophil elastase (NE) plays a major role in atherosclerosis. Bone marrow cells were harvested from both wild‐type (WT) and NE^−/−^/Apolipoprotein E (ApoE)^−/−^ double knockout (NE_KO) mice and transplanted into lethally irradiated WT and NE_KO male mice as indicated, respectively. After transplantation, mice were placed on a rodent chow diet for 4 to 5 weeks and then on a high‐fat diet for 12 weeks. Peripheral blood monocytes (A), aortas (B), and aortic roots (C through F) from 4 groups of transplanted mice were harvested and subjected to real‐time quantitative polymerase chain reaction analyses to detect NE mRNA expression levels (A), Oil Red “O” enface staining (B), hematoxylin‐eosin staining (C and D), collagen staining with Sirius Red (E), and Oil Red “O” staining (F), respectively. Respective representative image and quantitative data from 10 mice (n=10 mice per group) are presented. *P<0.05 (vs WT to WT group).

**Figure 9:**
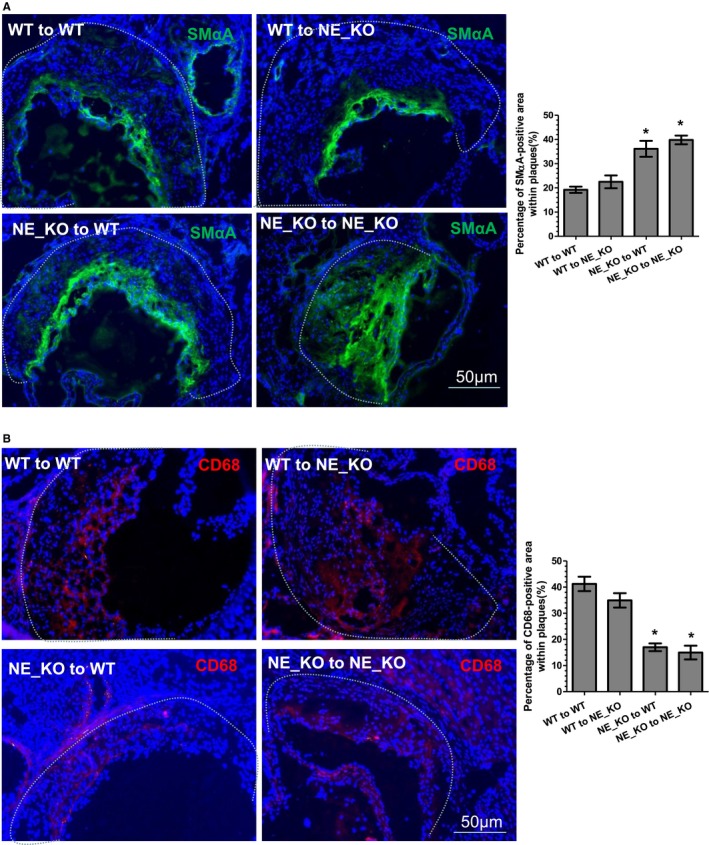
Smooth muscle cells and macrophage content in atherosclerotic plaques of the mice with bone marrow transplantation. Aortic roots from 4 groups of transplanted mice were harvested and subjected to immunofluorescent staining analyses using antibodies against smooth muscle α‐actin (SMαA) (A) and CD68 (B), respectively. Respective representative images and quantitative data (bar graphs) from 10 mice (n=10 mice per group) are presented. Dot lines indicate the boundary between media layer and atherosclerotic lesion. *P<0.05 (vs wild‐type [WT] to WT mice). NE_KO indicates neutrophil elastase^−/−^/Apolipoprotein E^−/−^ double knockout mice.

To elucidate the importance of NE in bone marrow cells and/or circulating inflammatory cells, BMTs between WT and NE_KO mice were conducted. Four to 5 weeks after BMT, recipient mice were subjected to atherosclerosis induction by feeding them an HFD for 12 weeks. Bone marrow reconstitution of recipient mice was confirmed by polymerase chain reaction analyses of NE mRNA expression level in peripheral blood monocytes. As expected, NE_KO mice that received WT bone marrow cell transplantation (WT→NE_KO) exhibited a comparable NE mRNA level to that of WT recipient mice (WT→WT), while NE mRNA was almost undetectable in the mice transplanted with NE‐deficient bone marrow cells (NE_KO→WT and NE_KO→NE_KO) (Figure 8A), confirming the success of the BMT procedure. Using these models, we observed that transplantation of NE‐deficient bone marrow cells to WT mice (NE_KO→WT) significantly inhibited atherosclerotic lesion formation to a comparable level to that of NE_KO mice that had received NE‐deficient bone marrow cells (NE_KO→NE_KO). In contrast, transplantation of NE_KO mice with WT bone marrow (WT→NE_KO) developed an advanced atherosclerotic lesion to a similar level to that of WT mice with WT bone marrow cell reconstitution (WT→WT), as demonstrated in vascular tree enface Oil Red “O” staining (Figure 8B). Such an effect was further confirmed in aortic roots (Figure 8C). Histological analyses of aortic roots with H&E (Figure 8D), collagen (Figure 8E), and Oil Red “O” (Figure 8F) staining revealed that the atherosclerotic plaques of 2 groups of mice with NE‐deficient bone marrow cell reconstitution (NE_KO→WT and NE_KO→NE_KO) had much smaller necrotic cores (Figure 8D) and a significantly higher collagen content (Figure 8E), but lower lipid accumulation (Figure 8F), than those of mice who received WT bone marrow cell transplantation (WT→WT and WT→NE_KO). Additionally, compared with mice that received WT bone marrow cell transplantation (WT→WT and WT→NE_KO), a much higher SMC (Figure 9A) but lower macrophage (Figure 9B) content was observed in atherosclerotic lesions of mice with NE‐deficient bone marrow cell reconstitution (NE_KO→WT and NE_KO→NE_KO). Collectively, these data demonstrate that bone marrow cell–derived NE is the major contributor to NE‐mediated atherosclerosis.

### Pharmacological Inhibition of NE Reduces Atherosclerosis in NE^+/+^/ApoE^−/−^ Mice

**Figure 10:**
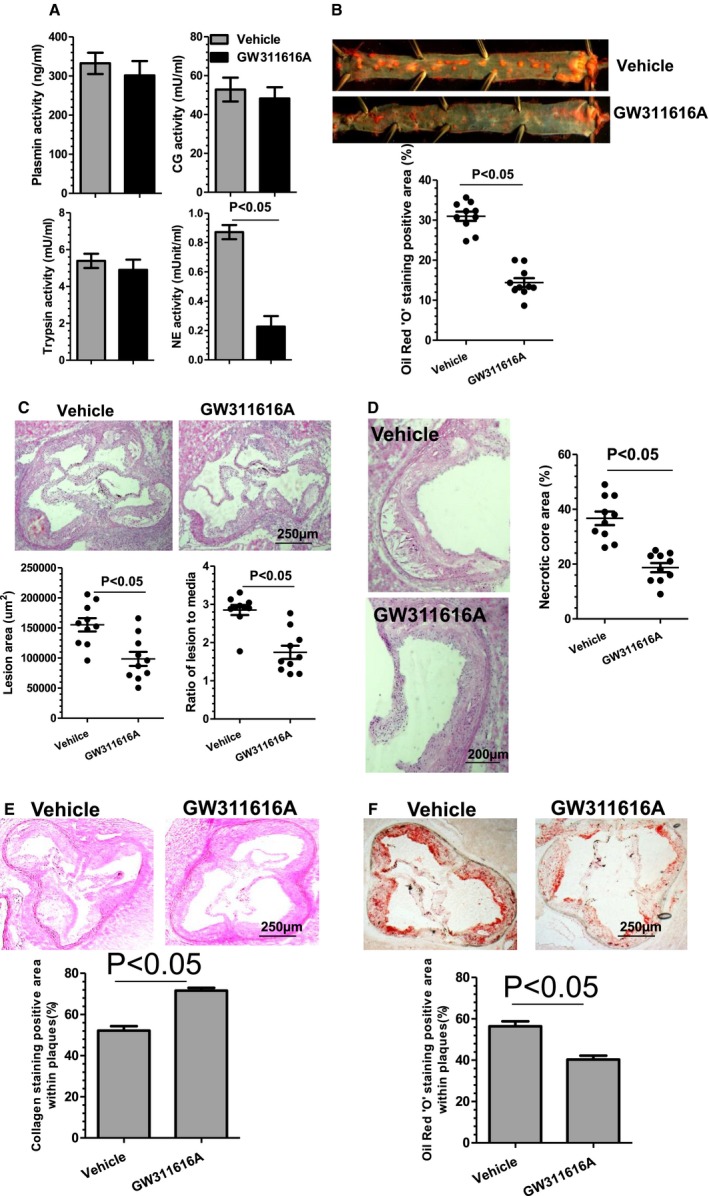
Neutrophil elastase (NE) pharmacologic inhibition reduces atherosclerosis in NE ^+/+^/Apolipoprotein E (ApoE)^−/−^ mice. Eight‐week‐old male wild‐type (WT) (NE ^+/+^/ApoE^−/−^) mice were fed a high‐fat diet (HFD) for 6 weeks, then randomly administrated vehicle or NE inhibitor (GW311616A, 2 mg/kg) twice a week through oral gavage for 6 weeks. All mice were continued to be fed with an HFD during drug administration. Plasma (A), aortas (B), and aortic roots (C through F) from these mice were harvested and subjected to plasma protease activity assays (A), Oil Red “O” enface staining (B), hematoxylin‐eosin staining (C and D), collagen staining with Sirius Red (E), and Oil Red “O” staining (F), respectively. Respective representative image and quantitative data from 10 mice (n=10 mice per group) are presented.

**Figure 11:**
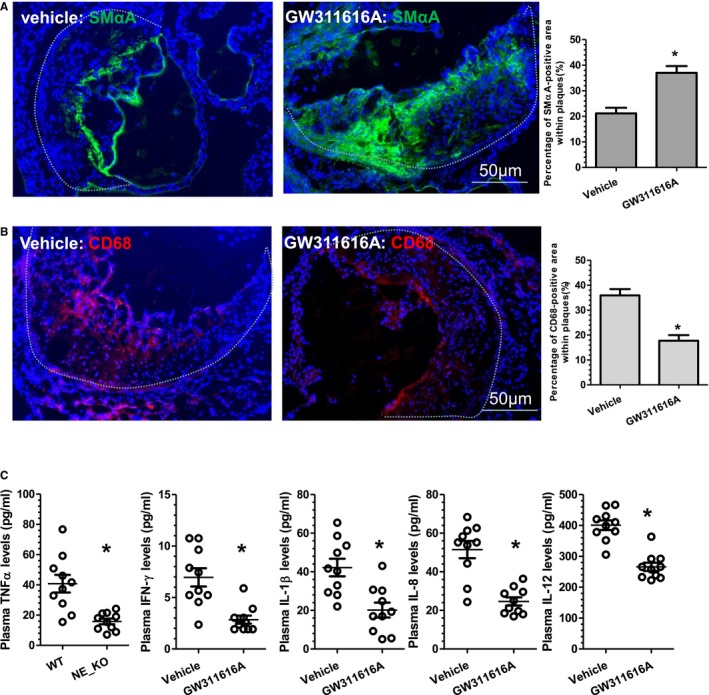
Neutrophil elastase (NE) inhibition by GW311616A increases indicators of atherosclerotic plaque stability. Aortic roots and plasma from the mice received similar treatments as described in Figure 10 were harvested and subjected to immunofluorescent staining analyses using antibodies against smooth muscle α‐actin (SMαA) (A) and CD68 (B) or plasma inflammatory cytokines (C), respectively. Respective representative images and quantitative data from 10 mice (n=10 mice per group) are presented. Dot lines indicate the boundary between media layer and atherosclerotic lesion. *P<0.05 (vs wild‐type [WT] mice). IFN‐γ indicates interferon γ; IL, interleukin; TNFα, tumor necrosis factor α.

To further explore the translational value of NE inhibition in prevention of atherosclerosis, a highly potent, selective, intracellular, orally bioavailable, and long‐duration HNE inhibitor, GW311616A,^15^ was orally administered to WT (NE^+/+^/ApoE^−/−^) mice. Since our findings showed that plasma NE activity is slightly increased during the early stage of atherosclerosis development (6 weeks of HFD feeding), but peaks at 12 weeks of HFD feeding (Figure 1B), GW311616A (2 mg/kg, twice a week) was administered from week 7 to 12 of HFD feeding. While GW311616A administration produced no significant effects on several proteases activity including CG, trypsin, and plasmin (Figure 10A), this protocol led to a significant inhibition of plasma NE activity (Figure 10A) and decreased size of atherosclerotic lesions as well as necrotic cores, as demonstrated in both vascular tree enface Oil Red “O” staining (Figure 10B) and aortic roots H&E staining (Figure 10C and 10D). Importantly, a higher level of collagen content (Figure 10E), but less lipid accumulation (Figure 10F), was observed within atherosclerotic lesions of the aortic roots of the GW311616A‐treated mice, compared with those of mice treated with vehicle control. Similarly, the atherosclerotic lesions of the aortic roots of the GW311616A‐treated mice contained increased numbers of SMCs (Figure 11A), but decreased numbers of macrophages (Figure 11B). Finally, significantly lower levels of plasma proinflammatory cytokines (eg, TNFα, IFN‐γ, IL‐1β, IL‐8, and IL‐12) were observed in mice administered GW311616A (Figure 11C).

### NE Promotes Foam Cell Formation In Vitro and In Vivo by Inhibiting Macrophage Cholesterol Efflux Capacity

**Figure 12:**
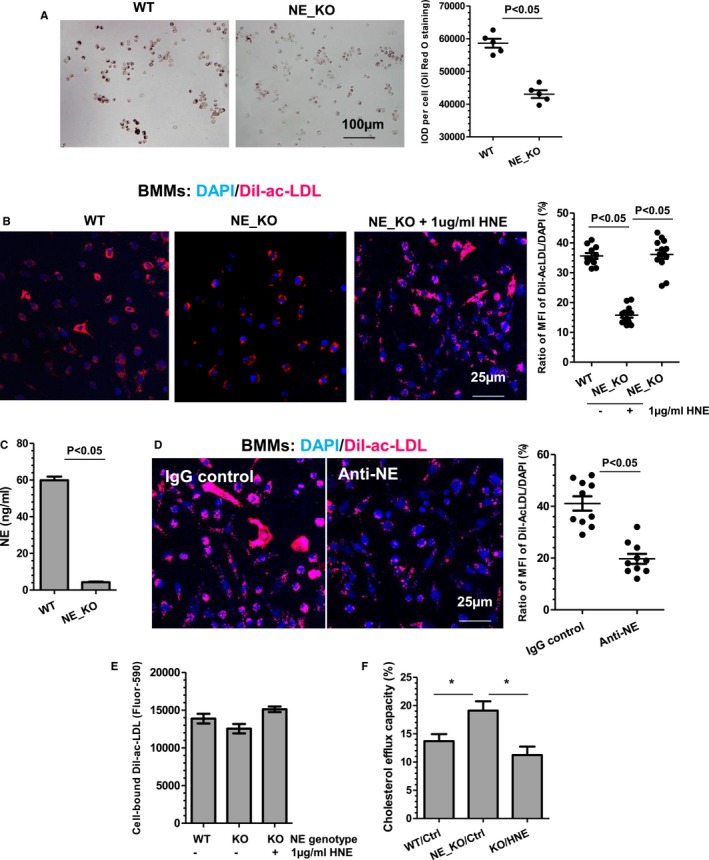
Neutrophil elastase (NE) promotes foam cell formation by inhibiting macrophage cholesterol efflux capacity. A, NE deficiency results in decreased foam cell formation in vivo. Peritoneal‐naive macrophages were isolated from wild‐type (WT) and NE^−/−^/Apolipoprotein E^−/−^ double knockout (NE_KO) mice fed a high‐fat diet for 12 weeks and subjected to Oil Red “O” staining. Representative foam cell image and quantitative data of the integrated optical density (IOD) per cell for Oil Red “O” staining are presented (n=5 mice per group). B, NE promotes foam cell formation from bone marrow–derived macrophages (BMMs). Bone marrow monocytes isolated from WT or NE_KO mice were induced to differentiate to BMMs by macrophage colony‐stimulating factor, followed by incubation with 5 μg/mL DiI‐Ac‐LDL in the absence or presence of 1 μg/mL active human neutrophil elastase (HNE; Abcam, ab91099) for 5 hours at 37°C. Cells were fixed and counterstained with 4′,6‐diamidino‐2‐phenylindole (DAPI). Representative foam cell image and quantitative data of the ratio of mean fluorescent intensity (MFI) of DiI‐Ac‐LDL (red) and DAPI (blue) from 10 to 12 images are presented. C, NE protein detection in BMMs conditioned culture medium. Serum‐starved WT and NE_KO BMMs were cultured in serum‐free fresh RPMI1640 medium for 24 hours. Cell supernatant was harvested and used for NE protein concentration measurement. D, NE secreted from BMMs promotes foam cell formation. NE_KO BMMs were incubated with WT BMMs conditioned culture medium in the presence of 1 μg/mL IgG or anti‐NE antibody for 6 hours, then subjected to foam cell formation assay as described in (B). E, NE plays no significant role in DiI‐Ac‐LDL binding to peritoneal‐naive macrophages. F, NE inhibits peritoneal naïve macrophage cholesterol efflux capacity. Peritoneal‐naive macrophages were isolated from WT and NE_KO mice and subjected to cholesterol efflux assays using 10 μg/mL Apolipoprotein A‐I as cholesterol acceptor. Data presented are an average of 5 independent experiments (n=5 in C, E, and F). *P<0.05 (WT vs NE_KO or HNE treatment vs vehicle control).

Foam cell formation is a key determinant of atherosclerosis formation and plaque phenotype. Since previous findings showed that the macrophage content within atherosclerotic lesions of aortic roots was significantly decreased in NE_KO mice or WT mice treated with GW311616A, we hypothesized that NE plays a role in foam cell formation. To address this, we first examined whether foam cell formation in vivo was altered by NE gene inactivation using a method described in previous studies.^24^, ^25^ Data shown in Figure 12A revealed that much less foam cell formation was observed in the peritoneal cavity of NE_KO mice fed an HFD for 12 weeks. A similar effect of NE gene inactivation on foam cell formation was observed with bone marrow–derived macrophages (Figure 12B). Importantly, the inhibitory effect of NE deficiency on foam cell formation was reversed by exogenous HNE (Figure 12B), collectively suggesting a critical role for NE in foam cell formation in vivo and in vitro. In addition, significantly higher levels of NE protein were detected in the culture medium of WT macrophages (Figure 12C), and a much lower level of foam cell formation was observed in NE_KO macrophages incubated with WT macrophage culture medium in which NE protein was depleted using an NE neutralizing antibody (Figure 12D). Collectively, these data show that NE is constitutively produced/secreted from macrophages and exerts its effects on foam cell formation through an autocrine manner.

To determine whether NE controls foam cell formation by modulating macrophage lipid binding and/or cholesterol efflux, these parameters were investigated using WT and NE_KO peritoneal‐naive macrophages. While no significant difference in lipid binding was noted between WT and NE‐deficient macrophages (Figure 12E), NE‐deficient macrophages exhibited an increased level of cholesterol efflux capacity compared with WT cells (Figure 12F). Importantly, the effect of NE gene inactivation on macrophage cholesterol efflux was abolished by addition of exogenous HNE (Figure 12F), confirming a key role for NE in macrophage cholesterol efflux and foam cell formation.

### NE Inhibits Macrophage Cholesterol Efflux by Promoting ABCA1 Protein Degradation in Macrophages

**Figure 13:**
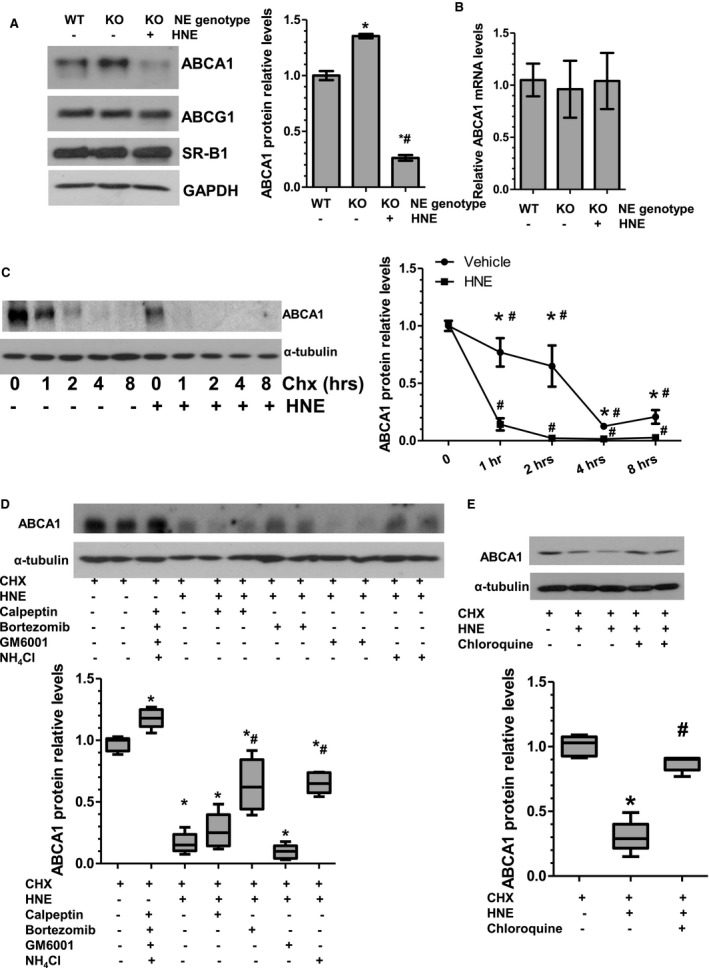
Neutrophil elastase (NE) promotes ABCA1 protein degradation in macrophages. A and B, ABCA1 protein but not the mRNA expression level in bone marrow–derived macrophages (BMMs) was inhibited by NE. Bone marrow monocytes isolated from wild‐type (WT) or NE^−/−^/Apolipoprotein E^−/−^ double knockout (NE_KO) mice were induced to differentiate to BMMs by macrophage colony–stimulating factor, followed by serum starvation overnight. Serum‐starved BMMs were treated with vehicle control or 1 μg/mL human neutrophil elastase (HNE) for 4 hours at 37°C. Protein and RNAs were harvested and subjected to Western blot (A) and real‐time quantitative polymerase chain reaction (B) analyses, respectively. Representative blots (A) and quantitative data of the relative ABCA1 protein (A) or RNA (B) expression levels from 5 independent experiments (n=5) are presented. *P<0.05 (NE_KO vs WT); ^#^ *P*<0.05 (HNE vs vehicle control). C, NE promotes ABCA1 protein degradation. NE_KO BMMs were incubated with vehicle control or 1 μg/mL HNE for 0, 1, 2, 4, or 8 hours in the presence of 10 μg/mL cycloheximide (CHX) as indicated. Representative blots and quantitative data of the relative ABCA1 protein expression levels from 5 independent experiments (n=5) are presented. *P<0.05 (HNE vs vehicle control at the indicated time points), ^#^ *P*<0.05 (vs 0 hour) (2‐way ANOVA test with Bonferroni post hoc test). D and E, NE promotes ABCA1 protein degradation, partially through lysosomal system. NE_KO BMMs were incubated with vehicle control or 1 μg/mL HNE for 1 hour in the presence of 10 μg/mL CHX, and in the absence or presence of various inhibitors as indicated. 40 μg/mL Calpeptin, a cell‐permeable calpain inhibitor; 100 nmol/L Bortezomib, a reversible inhibitor of the 26S proteasome; 10 μmol/L GM6001, a pan‐matrix metallopeptidases inhibitor; 2.5 mmol/L NH_4_Cl, a typical lysosomal inhibitor; and 100 μmol/L Chloroquine, a specific inhibitor of the lysosomal system. Data in (D and E) were representative blots and quantitative data of the relative ABCA1 protein expression levels from 5 independent experiments (n=5) presented as median with interquartile range. *P<0.05 (vs vehicle control); ^#^ *P*<0.05 (inhibitors vs vehicle control in the presence of HNE) (Kruskal–Wallis 1‐way ANOVA with a Dunn post hoc test).

To elucidate the underlying mechanism through which NE inhibits macrophage cholesterol efflux, we first examined whether the protein expression levels of the ATP‐binding cassette transporter A1 and G1 (ABCA1 and ABCG1) and scavenger receptor‐B1 (SR‐B1), 3 important cholesterol efflux regulatory proteins, were affected by NE in macrophages. Compared with that of WT macrophages, ABCA1 protein levels were increased in NE‐deficient macrophages (Figure 13A). Importantly, incubation of NE‐deficient macrophages with exogenous HNE significantly decreased ABCA1 protein expression. No significant difference between WT and NE‐deficient macrophages was observed with respect to the other 2 proteins, ABCG1 and SR‐B1. Furthermore, the expression levels of these proteins in NE‐deficient macrophages were not altered by exogenous HNE treatment (Figure 13A). Interestingly, ABCA1 mRNA level was not altered by NE gene inactivation in macrophages or incubation of NE‐deficient macrophages with exogenous HNE (Figure 13B), suggesting that NE inhibits ABCA1 protein expression through a posttranscriptional mechanism.

To further distinguish whether NE reduces macrophage ABCA1 protein expression through inhibiting ABCA1 protein translation or promoting ABCA1 protein degradation, an ABCA1 protein stability assay was conducted on NE‐deficient macrophages in the presence of cycloheximide, a protein biosynthesis inhibitor. Data shown in Figure 13C revealed that the half‐life of ABCA1 protein turnover was ≈3 hours in NE‐deficient macrophages, a response that was significantly reduced by exogenous HNE, indicating that NE promotes ABCA1 protein degradation.

To further uncover the underlying signaling pathways of NE‐mediated ABCA1 protein degradation, the potential effects of various protease inhibitors on NE‐mediated ABCA1 protein degradation in NE‐deficient macrophages were examined. We observed that the ABCA1 protein degradation mediated by NE was partially rescued by bortezomib (a specific inhibitor of the proteasome system), NH_4_Cl and chloroquine (both are inhibitors of the lysosomal system), but not by Calpeptin (a cell‐permeable inhibitor specific for calpains) and GM6001 (a pan‐matrix metalloproteinases inhibitor) (Figure 13D and 13E). Together these results infer a role for the lysosomal system in NE‐mediated ABCA1 protein degradation.

### NE Deficiency Leads to Increased ABCA1 Protein Expression but Less Lipid Loading in Lesion Macrophages

**Figure 14:**
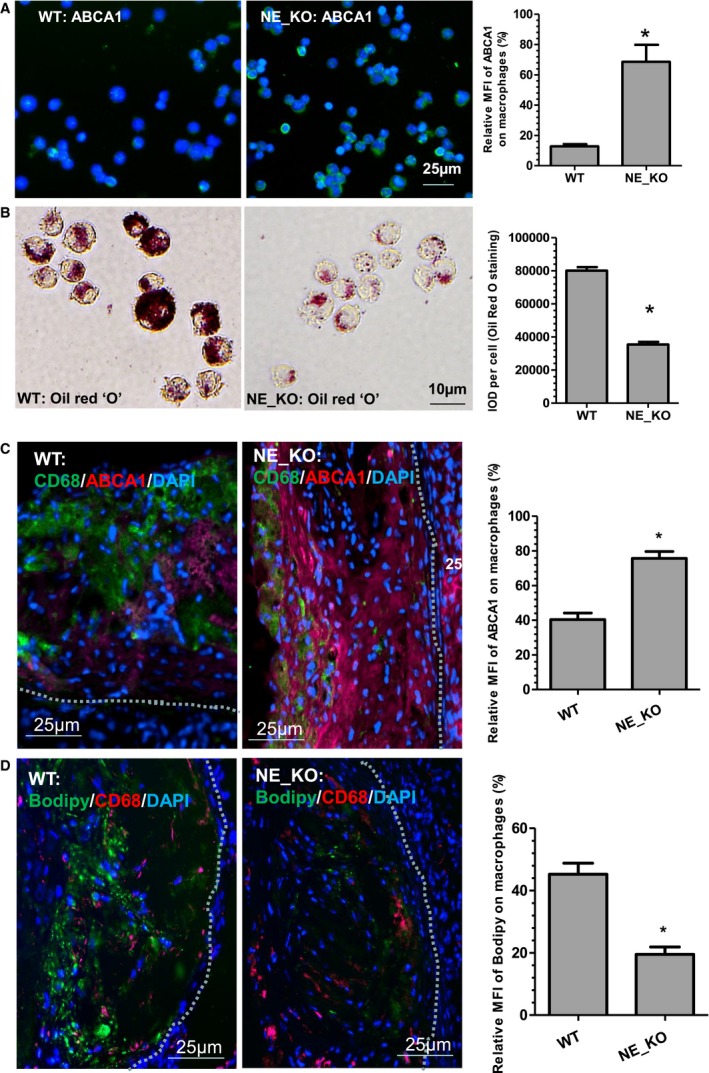
Neutrophil elastase (NE) deficiency leads to increased ABCA1 protein expression but less lipid‐loading in lesion macrophages. A, A higher level of ABCA1 protein expression was observed in macrophages isolated from the aortas of NE^−/−^/Apolipoprotein E^−/−^ double knockout (NE_KO) mice. Eight‐week‐old male wild‐type (WT) and NE_KO mice were fed a high‐fat diet for 12 weeks. CD68^+^ macrophages isolated from these mice were subjected to immunofluorescence staining with ABCA1 antibody. Representative images and quantitative data of the ratio of mean fluorescent intensity (MFI) of ABCA1 (green) and 4′,6‐diamidino‐2‐phenylindole (DAPI) (blue) from 10 images are presented. (B) NE‐deficient arterial macrophages were loaded with less lipids. Sorted arterial macrophages were subjected to Oil Red “O” staining. Representative images and quantitative data of the integrated optical density (IOD) per cell for Oil Red “O” staining are presented (n=10 images). C and D, Macrophages within atherosclerotic plaques of NE_KO mice expressed higher amounts of ABCA1 protein but were loaded with fewer lipids. Aortic roots sections obtained from the experiments described in Figure 4 were subjected to double immunofluorescence staining with antibodies against CD68 and ABCA1 (C), or CD68 antibody and Bodipy fluorescent dye (for staining lipids) (D), respectively. Representative images and quantitative data of the ratio of MFI of ABCA1 (red) and DAPI (blue) in (C), or the ratio of MFI of Bodipy (green) and DAPI (blue) in (D), from 10 mice (n=10 mice per group) are presented. *P<0.05 (vs WT).

Our in vitro data demonstrate that NE plays a key role in macrophage lipid accumulation and foam cell formation. To further explore whether NE possesses a similar effect on lesion macrophages during atherogenesis, lesion macrophages were isolated from the aortas of WT and NE_KO mice fed an HFD for 12 weeks and subjected to various analyses. We observed a much higher level of ABCA1 protein expression (Figure 14A), but less lipid accumulation (Figure 14B), in the arterial macrophages isolated from NE_KO mice, compared with that of arterial macrophages isolated from WT mice. Similarly, we observed that the ABCA1 protein was much more abundant in macrophages within atherosclerotic plaques of NE_KO mice (Figure 14C), while they had much less lipid loading (Figure 14D), compared with the macrophages within atherosclerotic lesions of WT mice. Collectively, these data support a role for NE in macrophage ABCA1 protein degradation and foam cell formation during atherogenesis.

## Discussion

As a multifunctional protease, NE has been reported to play a regulatory role in host defense by participating in the nonoxidative pathway of intracellular and extracellular pathogen destruction, and in noninfectious inflammatory diseases. As such, it has unsurprisingly been implicated in various human diseases including congenital neutropenia,^32^ chronic obstructive pulmonary disease, cystic fibrosis, acute lung injury, and acute respiratory distress syndrome.^1^, ^2^ Studies have also reported that elevated levels of plasma NE were observed in patients with various cardiovascular diseases,^33^, ^34^, ^35^, ^36^ and NE mRNA and protein were both detectable within human atherosclerotic plaques.^7^ More recent studies have reported increased plasma NE activity during atherosclerosis development^37^ and a reduction of atherosclerotic lesion in NE/proteinase 3 double knockout mice.^38^ Accordingly, NE has been suggested as a therapeutic target in atherosclerosis^39^ and could be used as a specific target for molecular imaging of early atherosclerotic lesions^37^; however, to date there has been no direct evidence indicating a causal role for NE in cardiovascular diseases. In the current study, we documented such a role for NE in atherosclerotic lesion formation. Our data show that inactivating the NE gene causes a substantial reduction in the extent of atherosclerosis in ApoE‐deficient mice fed an HFD. The atherosclerotic lesions in the NE_KO mice have smaller necrotic cores and less lipid accumulation but larger collagen content. Compared with controls, the atherosclerotic plaques in NE_KO mice have higher SMC but lower leukocyte (including neutrophil and macrophage) content, likely reflecting the role of NE in increasing the indicators associated with a vulnerable plaque phenotype. As expected, data from BMT revealed that bone marrow cell–derived NE is the main contributor to NE‐mediated atherogenesis. Translationally, we found that oral administration of a pharmacological NE inhibitor into ApoE‐deficient mice significantly reduced atherosclerotic lesion size. Taken together, these results indicate that NE plays a causal role in atherogenesis.

Our findings are consistent with observations reported in various previous preclinical^37^, ^38^ and clinical^7^, ^33^, ^34^, ^35^, ^36^ studies that suggest an important role for NE in atherogenesis; however, these observations are disputed in another study. Soehnlein et al^40^ reported that mice lacking both NE and proteinase 3 displayed reduced atherosclerotic lesion size at early but not at advanced stages of lesion development, whereas lack of NE alone did not affect lesion formation. Such a discrepancy may be attributable to differences in experimental settings and parameters used for evaluating atherosclerotic lesions in these studies. However, it is difficult for us to directly compare our findings with their observation, since crucial information concerning the mice used in their study (eg, age, sex, animal's genetic background, and the composition of the HFD) were not available. Moreover, no evidence confirming the absence of NE in the NE genetically modified model (eg, NE mRNA, protein expression, or enzymatic activity) was reported in their study. Furthermore, their measurements used for reporting the atherosclerosis were different from ours, which may also explain the discrepancy. For instance, only atherosclerotic lesion size (percentage of aortic root area) was used by Soehnlein et al, while a variety of well‐established atherosclerotic lesion parameters were used in our study, including Oil Red “O” staining for the positive area (%) within the whole arterial tree and the aortic roots, and the lesion area (μm^2^) and ratio of lesion area to medial area. Finally, the duration for which the mice were fed the HFD between 2 studies was also different (eg, 4 weeks and 4 months in Soehnlein's study, compared with 12 weeks in our study), which may also suggest a spatial and temporal effect of NE on atherogenesis.

We observed that NE is mainly found in the ECM within atherosclerotic plaques, while the atherosclerosis‐related cells contain a variety of amounts of NE (Figure 1C through 1F and Figure 2). Such unexpected observations may be attributable to the following: (1) the cells within atherosclerotic plaques including leukocytes, macrophages, vascular SMCs, and endothelial cells produce no or a low level of NE; (2) they do synthesize de novo NE, but rapidly release it into the surrounding ECM; and (3) they do not synthesize de novo NE, but internalize NE that is assimilated into the plaque from degranulating neutrophils during their passage through the rich plaque neointimal vasculature. Regardless, the ECM localization of NE within atherosclerotic plaques may represent a mechanism through which NE promotes atherogenesis. It is known that NE can mediate ECM proteolysis and exert other physiological and pathological functions, despite the presence of a high level of endogenous NE inhibitors. Although the exact mechanisms are still debatable, growing evidence has suggested that the large quantities of oxidants and proteases released by leukocytes that are recruited to sites of inflammation can overwhelm and inactivate protease inhibitors. Furthermore, a large proportion of the serine proteases that have been released from cells can bind to plasma membranes and/or ECM with their catalytic activity preserved, which makes them inaccessible, and therefore resistant, to circulating high‐molecular‐weight endogenous inhibitors.^41^ Therefore, NE present within atherosclerotic plaques in a compartmentalizing manner with a higher local concentration may contribute to the process of matrix degradation and the weakening of the vessel wall, which, in turn, is responsible for the atherosclerotic lesion formation.

One potential underlying mechanism through which NE promotes atherogenesis is that NE modulates inflammation. We found that, compared with controls, systemic and local aortic inflammation was diminished in NE‐deficient mice, as evidenced by NE deficiency causing a decreased level of circulating proinflammatory cytokines and a significant lower mRNA expression level of proinflammatory genes within the aortas (Figure 6B and 6C). In addition to its established roles in the degradation of the ECM proteins, accumulating evidence has pinpointed NE as a versatile mediator that fine‐tunes the systemic and local immune response and/or inflammation. It has been well summarized that NE regulates systemic and local immune responses and/or inflammation, through multiple mechanisms including: (1) promoting the release of active cytokines from their inactive precursor molecules; (2) increasing proteolytic cleavage, and thus inactivation of active cytokines; (3) enhancing proteolysis of cell surface‐bound cytokine receptors or cytokine binding proteins; and (4) activating specific cell surface receptors (such as protease‐activated receptors or toll‐like receptor 4).^42^, ^43^ Specifically, NE has been reported to modulate the activity of important regulators controlling inflammatory processes (eg, TNFα, IL‐6, IL‐8, SDF1α, MIP1α, chemerin, and granulocyte‐colony stimulating factor), adaptive immune responses (eg, IL‐2, chemerin, CD2, CD4, and CD8), or the repair processes (eg, TGF‐β, TGF‐α, epidermal growth factor, insulin‐like growth factor, TNFα, TNF‐RII, and proepithelin).^42^, ^43^ We found that both the circulating and aortic expression levels of multiple proinflammatory cytokines (eg, TNFα, IFN‐γ, IL1β, IL‐8, and IL‐12) were significantly decreased in NE‐deficient mice. Such findings indicate that NE promotes atherogenesis through activating these proinflammatory cytokines, which are consistent with the regulatory role of NE in immune responses and/or inflammation. However, it is worth noting that NE regulation of the inflammatory response is cell content dependent. Different studies showed that NE increases IL‐8 in endothelial cells,^44^ while NE suppresses IL‐8 expression in SMCs.^45^ Evidence has also suggested that NE may function as a negative regulator of inflammation. Several studies^46^, ^47^, ^48^ have shown that NE is capable of degrading numerous proinflammatory cytokines such as IL‐1β, TNFα, IL‐2, and IL‐6, further indicating that NE possesses a divergent role in immune response and/or inflammation.

A new finding from the current study is that NE promotes foam cell formation during atherogenesis. Our data show that NE regulates macrophage cholesterol efflux capacity but not its binding ability to lipids. Several lines of evidence^5^, ^49^ suggest that NE can modify LDLs and enhance their uptake by macrophages through the LDL receptor pathway. However, no significant difference in DiI‐Ac‐LDL binding to macrophages was observed between the macrophages isolated from WT and NE‐deficient mice. This is not surprising since there was no significant difference in the protein expression levels of LDL receptor observed between WT and NE‐deficient macrophages (data not shown). In contrast, we have obtained clear evidence for the ability of NE to increase lipid accumulation in macrophages through inhibition of cholesterol efflux, an effect that provokes foam cell formation. In addressing the mechanisms through which this occurs we have provided evidence for the ability of NE to promote foam cell formation through modulating ABCA1 protein degradation. Foam cell formation occurs mainly as a result of an imbalance between lipid binding/uptake and efflux in macrophages, which results in excessive lipoprotein‐derived cholesterol accumulation inside macrophages. It is well‐known that lipid binding/uptake is mediated by various receptors including SR‐A and CD36,^50^, ^51^ while the efflux of accumulated cholesterol in macrophages is mainly controlled by reverse cholesterol transporters including SR‐BI,^52^ ABCA1^53^ and ABCG1.^54^ Our data show that the ABCA1 protein, but not the SR‐BI and ABCG1 proteins, is altered in NE‐deficient macrophages or affected by addition of exogenous HNE, indicating that ABCA1 is selectively responsible for NE‐promoting foam cell formation. More importantly, our data further demonstrate that ABCA1 protein degradation, but not its transcription and translation, was altered by NE gene inactivation or exogenous HNE treatment. Currently, 2 major signal pathways have been implicated in ABCA1 protein degradation: PEST sequence‐mediated calpain proteolysis^55^ and the proteasome‐lysosome system.^56^, ^57^ We found no evidence to suggest that the calpain pathway is the underlying mechanism of NE‐promoted proteolysis of ABCA1, since NE‐mediated ABCA1 protein degradation was not affected by calpain‐specific inhibition. Moreover, it seems that the proteolysis pathway mediated by matrix metalloproteinases is not responsible for NE‐mediated ABCA1 proteolysis, as evidenced by the finding that NE‐mediated ABCA1 protein degradation process was not impaired by the addition of the matrix metalloproteinase inhibitor GM6001. On the other hand, the promotive effect of NE on ABCA1 protein turnover was partially corrected by bortezomib, NH_4_Cl, or chloroquine, confirming a role for the lysosomal system in NE‐mediated ABCA1 proteolysis.

Data with proteolysis inhibitors showed that inhibiting the proteasome‐lysosome system could not completely reverse the promotive effect of NE on ABCA1 proteolysis. This suggests that additional proteolysis signaling pathway(s) may also participate in NE‐mediated ABCA1 protein degradation, which warrants further study in the future. Although there is no reliable animal model for plaque rupture, the potential effects of NE deficiency on plaque rupture remains to be explored. Finally, although NE has been detected in human fibrous and atheromatous plaques,^7^ our understanding of the functional role of NE in human atherogenesis requires further exploration.

Another interesting finding in the current study is that we observed significantly decreased levels of proinflammatory monocytes in both bone marrow and peripheral blood of NE_KO mice (Figure 7), indicating that NE plays a role in monocytosis and/or monopoiesis. Emerging evidence from animal and human studies has suggested that monocytosis can be an indicator of, and a key contributor to, various inflammatory diseases including atherosclerosis.^58^, ^59^, ^60^ Proinflammatory monocytes selectively migrate to the sites of inflammation (or dysfunctional endothelium) where they rapidly infiltrate into arterial wall and differentiate into inflammatory macrophages, producing inflammatory cytokines and contributing to local and systemic inflammation, which, in turn, promotes atherosclerosis.

Interestingly, seminal studies have indicated that cholesterol efflux pathways (eg, ABCA1/ABCG1) are the novel regulators of monopoiesis, inflammation, and atherogenesis.^61^, ^62^, ^63^, ^64^, ^65^ Transgenic studies revealed that ABCA1/ABCG1 gene–deficient mice fed an HFD had an expanded pool of hematopoietic stem/progenitor cells, resulting in monocytosis by directly producing blood monocytes or mobilizing to the spleen and initiating extramedullary hematopoiesis.^66^ Increased circulating monocytes enter the atheroma, differentiate into macrophages, and promote atherogenesis.^66^ A further study has identified an important role for ABCA1/ABCG1 in hematopoietic stem/progenitor cell mobilization/proliferation,^67^ which likely represents the underlying mechanism through which ABCA1/ABCG1 regulates monopoiesis. Apart from their critical role in monopoiesis, cholesterol efflux pathways have also been implicated in macrophage functions (eg, migration^68^ and foam cell formation^69^), and deficiency of ABCA1/ABCG1 in macrophages increases inflammation and accelerates atherosclerosis or atherosclerotic plaque growth.^70^, ^71^ In line with these important findings, we observed that mice defective in NE fed an HFD displayed a decreased monocytosis, low number of macrophages or foam cells within atherosclerotic plaques, smaller atherosclerotic lesion size, and a stable plaque phenotype, which may be mainly attributed to an increased ABCA1 protein level in NE‐deficient macrophages. Therefore, we propose that NE promotes atherogenesis by degrading ABCA1 protein, resulting in increased monocytosis, macrophage migration, and foam cell formation, which, in turn, leads to a higher level of local and systemic inflammation, and eventually increases atherosclerosis. Additionally, we also observed an increased level of ABCA1 protein expression in endothelial cells within atherosclerotic plaques in NE_KO mice (data not shown), which may also contribute to the decreased atherogenesis observed in these mice. These observations complement findings reported in a recent study looking at endothelial cells. Westerterp et al^72^ demonstrate that deficiency of ABCA1/ABCG1 in endothelial cells accelerates atherosclerosis, and they suggested that endothelial cell–derived ABCA1/ABCG1 plays a role in preservation of endothelial NO synthase activity and suppression of endothelial inflammation.

## Conclusions

In this study we provided several lines of direct evidence to support a causal role for NE in atherogenesis. Moreover, our data demonstrate that modulating systemic and local aortic inflammation, together with promoting foam cell formation by increasing ABCA1 protein degradation and reducing macrophage cholesterol efflux, are 2 major underlying mechanisms through which NE promotes atherosclerotic lesion formation. Our findings therefore indicate that pharmacological inhibition of NE may represent a potential therapeutic approach to treat cardiovascular diseases. Thus, our current study provides a strong rationale and paves the way for clinical studies using NE inhibitors in the treatment of atherosclerotic disease.

## Sources of Funding

This work was supported by British Heart Foundation (FS/09/044/28007, PG/11/40/28891, PG/13/45/30326, PG/15/11/31279, PG/15/86/31723, and PG/16/1/31892 to Xiao), the National Funds for Developing Local College and Universities (B16056001 to Wen), and the Wellcome Trust (098291/Z/12/Z to Nourshargh). This work forms part of the research portfolio for the National Institute for Health Research Biomedical Research Centre at Barts.

## Disclosures

None.
